# MinION Whole-Genome Sequencing in Resource-Limited Settings: Challenges and Opportunities

**DOI:** 10.1007/s40588-022-00183-1

**Published:** 2022-11-17

**Authors:** Fredrickson B. Wasswa, Kennedy Kassaza, Kirsten Nielsen, Joel Bazira

**Affiliations:** 1 Department of Microbiology and Parasitology, Mbarara University of Science and Technology, Mbarara, Uganda; 2 Department of Microbiology and Immunology, University of Minnesota, Minneapolis, MN USA

**Keywords:** Whole-genome, Sequencing, MinION, Flow cells

## Abstract

**Purpose of Review:**

The introduction of MinION whole-genome sequencing technology greatly increased and simplified complete genome sequencing in various fields of science across the globe. Sequences have been generated from complex organisms to microorganisms and are stored in genome databases that are readily accessible by researchers. Various new software for genome analysis, along with upgrades to older software packages, are being generated. New protocols are also being validated that enable WGS technology to be rapidly and increasingly used for sequencing in field settings.

**Recent Findings:**

MinION WGS technology has been implemented in developed countries due to its advantages: portability, real-time analysis, and lower cost compared to other sequencing technologies. While these same advantages are critical in developing countries, MinION WGS technology is still under-utilized in resource-limited settings.

**Summary:**

In this review, we look at the applications, advantages, challenges, and opportunities of using MinION WGS in resource-limited settings.

## Introduction

**Fig. 1:**
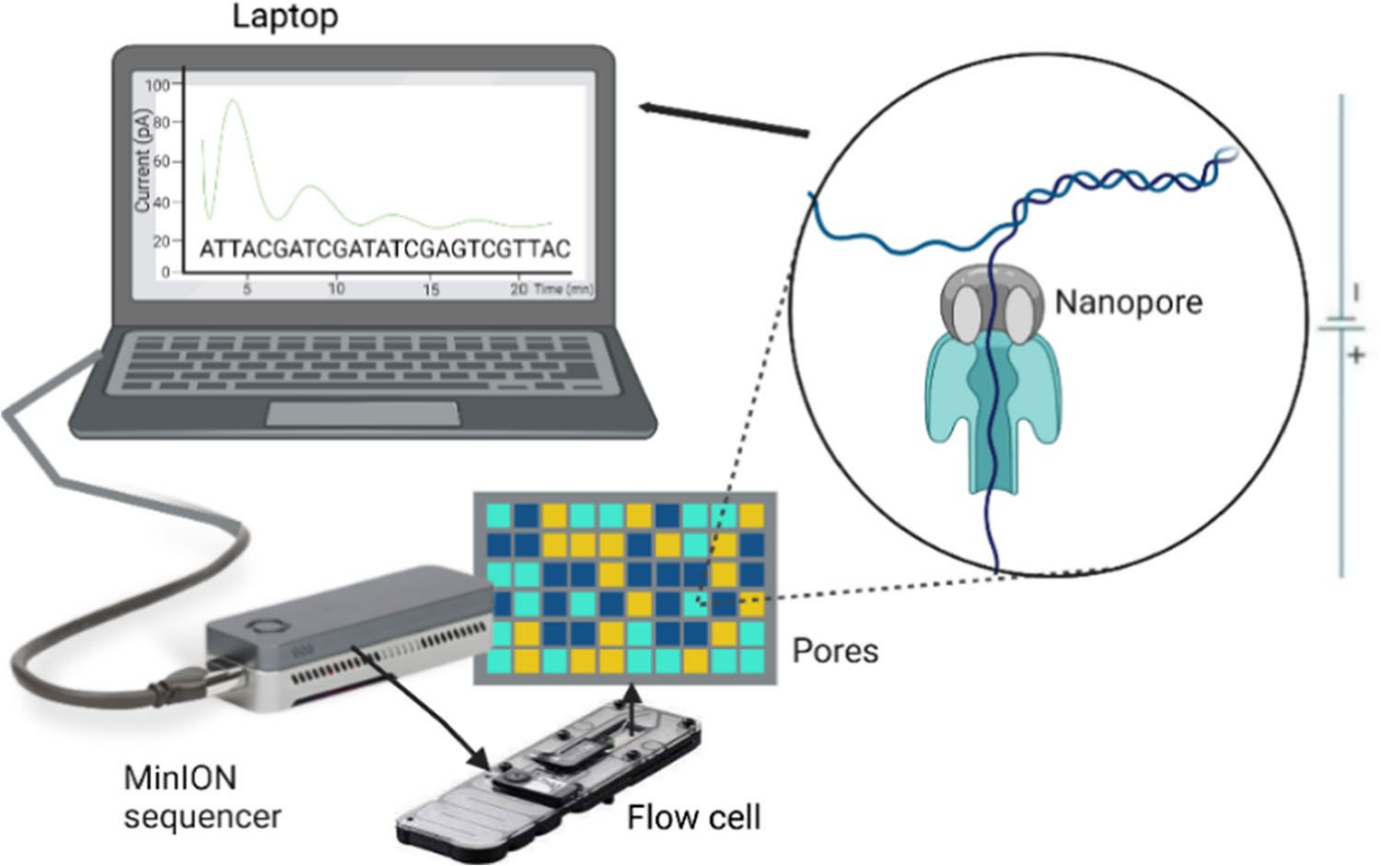
Schematic diagram indicating the composition of the MinION device, flow cell, nanopore, real-time computer access, and process management. Green is active, blue is inactive, and yellow are recovery pores on the nanopore membrane. The figure was designed using biorender [10]

The desire to understand human genetics led to a revolution in gene sequencing technologies [1]. As part of this sequencing revolution, Oxford Nanopore Technologies (ONT) invented the MinION whole-genome sequencer device (Fig. 1), which was released to the first commercial users in 2014 [2, 3]. MinION and similar sequencing technologies are classified as third-generation sequencers (TGS) because these technologies sequence single molecules of DNA and RNA directly, without a PCR amplification step [4]. The MinION has another added advantage over second-generation sequencers—it can sequence long DNA molecule lengths [5]. In addition, the MinION sequencer is portable, weighing less than 100 g, and can be connected to laptop or benchtop computers via a USB port. Real-time data analysis is obtained for both the number of sequence reads and the distribution of DNA lengths; thus, the system requires limited computing infrastructure [6, 7]. The technology uses a “flow cell” that consists of up to 2048 individual nanopores that are monitored in 512 separate groups by a microchip known as the application-specific integrated circuit (ASIC) [2, 8]. The device is equipped with specialized software, known as MinKWON, that is run on the computer to which the device is connected. The MinKWON software performs several functions including data acquisition, real-time analysis and feedback, data streaming (including run parameter selection), and sample identification and tracking. These processes allow the user to ensure the platform chemistry performs well while sequencing and to make real-time adjustments [8]. The read length of MinION WGS continues to be improved, with entire megabase chromosomes able to be sequenced as a single molecule, with each flow cell capable of generating hundreds of thousands of reads [9].

With the continued development of sequencing chemistry and software improvements, the capacity of MinION WGS has improved. MinION WGS has lower base call accuracy during the sequencing reaction than first- (Sanger sequencing) and second-generation (short-read WGS) sequencing [11, 12]. However, various software updates that bioinformatically account for base miscalls have improved the MinION WGS sequencing accuracy to 90% [9]. Many new software packages are being developed and validated [13, 14] that, combined with enhanced chemistry [15], are anticipated to improve the technology further.

One unique aspect of the ONT MinION sequencing platform is the emphasis on a low-cost, user-friendly system that allows for easy library preparation and consistent sequencing results [16]. This user-friendly focus reduces intellectual barriers for use, allowing researchers to readily perform sequencing without the support of core sequencing facilities. The technology is less expensive and more rapid compared to previous sequencing technologies [17]. Thus, the ONT MinION sequencing device has a wide range of applications that include de novo genome assembly [18] and classifications [19], identification and differentiation of bacteria and viruses [20], metagenomic analysis of microbes [21–24], real-time diagnostics [25, 26], on-site sequencing in extreme environments [16], and diagnosis of fungal pathogens [27–30].

This review will focus on how MinION WGS is utilized in resource-limited settings in the field of microbiology, the opportunities this technology creates in resource-limited settings, and the challenges that need to be overcome to obtain sequencing results.

## Principle of MinION WGS

The primary component of the MinION WGS is the membrane that contains nanopores (microscopic pores) to which current is applied (Fig. 1) [31]. The nanopore allows a single DNA or RNA molecule to pass through the membrane in the presence of an electrical current and appropriate buffer solutions. When the DNA/RNA passes through the nanopore, each nucleotide influences the current in a different way, which is detected by a sensor. These current changes are then decoded by the computer software, producing a sequence for the DNA/RNA strand as it passes through the pore [2, 8, 14]. Because sequencing occurs continuously for each DNA/RNA strand that passes through a pore, the only limitation to the length of DNA/RNA that can be analyzed is the user’s ability to obtain intact, long fragments of DNA from their cell of interest.

## Why Sequence in Resource-Limited Settings?

The sequencing needs in resource-limited settings are similar to other regions. For example, in the context of disease epidemiology, sequencing data generated on-site can be used in surveillance investigations to monitor for new isolates, the prevalence of existing isolates, and drug resistance during outbreaks [32, 33•, 34]. The ability to rapidly analyze sequencing information in order to adapt healthcare practices and best utilize limited resources is perhaps even more important in resource-limited settings than in wealthy countries where abundant healthcare and state-of-the-art treatments are readily available. In resource-limited settings, sequencing technology needs to be inexpensive and accessible to be useful.

The small size of the MinION WGS device and its accessories is advantageous in countries with limited resources where laboratories are small. There is no need for lab expansion or modifications before installation [35, 36]. Importantly, results can be obtained within one to a few hours, making the MinION device suitable for diagnostic purposes [36–39] and therefore providing real-time analysis of results. The use of battery power to overcome lack of electricity or routine electricity shortages is also easy due to the minimal power requirements when compared to other sequencers [40••]. While training is still required, the MinION device is user-accessible and requires less overall training and less technical expertise, making it accessible to a general user [36]. In addition, the initial equipment and reagent costs necessary to perform sequencing is substantially lower than other sequencing technologies [41, 42] and the sequencing cost per sample is also significantly lower. For example, estimated sequencing costs for MinION versus Illumina sequencers are £61.17 and £205.03, respectively [40••]. Furthermore, the MinION device comes with free software for the analysis of sequence data [36]. Although MinION results still have biases, technology upgrades are in place to ensure reliability and high-quality results [37]. Importantly, the use of MinION removes the challenges associated with shipping samples. Sample degradation and loss that would be encountered during shipping are reduced since the samples are either processed in the field or an on-site lab [37]. Expenses encountered in sending samples to established countries with sequencing facilities are minimized, as well as challenges associated with transport of highly infectious samples [37].

Because of advances revolutionized by the relatively cheap MinION sequencing technology compared to other infrastructure-intensive sequencing platforms, it is now possible to envision the research potential of employing MinION sequencing in resource-limited settings [40••]. For example, due to the portability of MinION sequencers [35], they were critical during the Ebola outbreak in Northern Africa in 2016 [39], given that Ebola outbreaks typically occur in locations where it is difficult to utilize other more bulky sequencers. Furthermore, the ability to perform on-site sequencing solved the challenge of transporting highly infectious Ebola samples to countries with established sequencing facilities. In addition, MinION sequencing is an excellent tool for epidemiological surveillance in resource-limited settings within and outside Africa for monitoring of disease outbreaks and drug resistance surveillance [32, 39, 43•, 44•]. MinION sequencers are able to detect mixed infections in a sample, which gives it an added advantage when used for diagnostic purposes [37].

Beyond disease monitoring, MinION sequencing is also used for population genetic studies in humans, animals, agriculture, and veterinary science under resource-limited conditions [36, 38, 40••]. Most human genome sequencing performed to date has occurred in wealthy societies [45–47]. Yet analysis of human genomes from different peoples is needed to identify the entirety of genetic diseases in humans [48–52]. The ability to transition from research of sequence data to clinical diagnosis in patients is a necessity across the globe [53]. The same concept applies to plants and microbes [54–56]. Sequencing technologies are used in a wide variety of biological applications ranging from agronomy, biochemistry, forestry, genetics, horticulture, pathology, and systematics [57, 58]. Importantly, Boykin et al. [38] were able to detect latent viruses in crop materials, gaining an added advantage over other sequencing technologies. Virus-indexing for the safe movement of germplasm can limit the movement of infected plant materials across the globe, resulting in improved international trade [37].

While MinION WGS technology is only just beginning to be implemented in resource-limited settings, the potential to revolutionize research in these settings and gain understanding of global diversity is becoming readily apparent. In Tanzania, MinION WGS was used in the study of Peste des petits ruminants virus which causes a contiguous disease in wild and small domestic ruminants and the researchers in this study were able to generate results within 4 h of sample collection [36]. In another study conducted in Uganda, Kenya, and Tanzania to identify viruses that cause disease in crops, results were obtained using MinION technology in 3 h compared to the typical 6 months. This study also demonstrated the use of PDQeX DNA purification technology, which does not require extensive infrastructure, highlighting the use of MinION sequencing in field activities [38]. In West Africa, Quick et al. demonstrated MinION WGS results could be obtained within 15–60 min of Ebola sample collection, showing its utility in surveillance and epidemiological investigations [39], while in DRC, another study considered genetic variabilities in order to predict future outbreaks of Ebola in West Africa [59•]. In a collaborative study conducted in Kenya and other countries outside Africa, the limitations of direct MinION sequencing for rabies virus were also highlighted, with known positive samples only detected by MinION when Ct values ranging from 14.4–27.1 showing PCR is more sensitive than MinION sequencing in the diagnosis of rabies [60••], while in another collaborative study in Kenya, Tanzania, the Philippines, and the UK about rabies, a consensus coverage for whole genome in all study sites was ≥ 20 × which can be used to handle outbreaks [40••]. In contrast, studies conducted in Indonesia show that MinION can be used to identify and monitor dengue virus in clinical samples and to monitor the clades currently circulating [42]. Finally, during the ongoing COVID-19 pandemic, MinION WGS was used for surveillance in Equatorial Guinea and was used to detect two variants of the beta and delta variants in a single asymptomatic patient [43•].

Perhaps most promising is the recent use of MinION WGS in infectious diseases research. MinION WGS proved to be a cheaper and quicker method for the diagnosis of nosocomial tuberculosis infections among children in an endemic region of Zambia, when compared to second next-generation Illumina sequencing technology [61]. In West Africa, MinION WGS enabled complete sequencing of the outbreak strain of *Neisseria meningitidis* that causes meningococcal and when compared to existing strain enabled to track the commensal strains which became a pathogen by stepwise acquisition of virulence factors [62], while in another study of the same organism by the same author in Ethiopia, MinION WGS was used along with Illumina technologies and predicted how the ST-192 clone could evolve to pathogen and how it would acquire a type B capsule, fetA, and infection with the MDAΦ phage although less likely would it become a pathogen [63]. A surveillance study conducted in West Africa on cholera outbreaks found no new *V. cholerae* O1 was introduced into the region from outside of West Africa between 2014 and 2018. Instead, population genetic analyses suggested nearby countries, like Ghana and Togo, had outbreaks that were genetically connected to outbreaks that occurred in Cameroon, Niger, and Nigeria [32]. In a study conducted in Kenya on *Neisseria gonorrhoeae* drug resistance, MinION WGS was able to detect strain drug resistance profiles, without the need for another sequencing platform like Illumina, allowing MINION WGS to be used for clinical diagnosis and epidemiological studies [64••]. In Ethiopia, a study focusing on the antimicrobial activity of *Streptomyces* spp. identified 36 biosynthesis gene clusters (BGCs) in the genome [65]. MinION WGS was used to identify *Paenibacillus* spp. among patients with hydrocephalus in Uganda and a complete genome sequence of *Paenibacillus thiaminolyticus* was generated by combining short-read sequencing, optical mapping (Bionano Genomics), and MinION sequencing [66, 67]. In Malawi, a genomic epidemiology study was conducted on *E. coli* using MinION sequencing to identify most drug-resistant sequence types [44•].

In other studies, MinION WGS was used in a resource-limited setting to identify human genetic factors underlying disease. Sickle cell disease and other hemoglobinopathies were studied using MinION WGS in Tanzania, which found the IVS1G > A mutation is present in clients of Arabic descent. MinION sequencing results were obtained from whole blood on the same day, compared to the traditional dried blood spot (DBS) used for sickle cell disease diagnosis. Furthermore, MinION sequencing of blood presents another advantage over DBS because it readily identifies incomplete Hb switching or co-inheritance of other hemoglobin variants [68]. MinION WGS was also shown to be equivalent to Sanger sequencing of the 16S and CO1 genes to identify similarities and differences between the tropical vertebrates *Amietophrynus brauni*, *Leptopelis vermiculatus*, *Rieppeleon brachyurus*, *Sorex alpinus*, *Arthroleptis xenodactyloides*, *Rhynchocyon udzungwensis*, and *Leptopelis vermiculatus* in Tanzania [41].

MinION sequencing has been successfully applied in the context of fungal detection in the developed world for various applications such as de novo genome assemblies of yeasts like *Saccharomyces cerevisiae* [69, 70], genome assembly from PCR products [29], detection of fungi using metagenomics [71, 72], fungal ecology studies [73], epidemiological outbreaks of Candida species [74], and diagnosis of fungal pathogens in plants [27, 28, 30]. However, in resource-limited settings, application of MinION sequencing technologies has not been yet applied, thus providing opportunities to explore the utilization of this technology in mycology.

## Challenges of Using MinION Sequencing in Resource-Limited Settings

Power blackouts are still a common challenge in resource-limited areas and can impact both MinION runs, storage conditions for reagents, and upload of data to cloud-based servers for data analysis [37, 40••]. Cutting-edge computers and accessories are not easily accessible in resource-limited areas, especially for field studies located in remote areas [37]. Shipping of disposable reagents from the manufacturer to resource-limited regions of the world is still a significant challenge, often due to the necessary reagent cold-chain transport requirements that cannot be guaranteed, resulting in ineffective or sub-optimal flow cells upon arrival. Maintaining proper reagent storage conditions in remote sites can also be challenging [37]. While the initial cost to establish MinION sequencing, and individual sample costs, is low compared to other sequencing platforms, MinION sequencing technology is still cost-prohibitive for many researchers and clinicians in low-resource settings [37]. The MinION WGS sequencing technology relies on internet connections with at least 3G capacity, which is still not available in some countries or regions, limiting on-site disease surveillance and outbreak investigations in these areas [37]. RNA degradation in stored samples requires stabilizing reagents that are often not accessible in low-resource settings, limiting the ability to collect samples for future surveillance and epidemiologic studies [40••]. Most laboratories have limited spaces from construction designs that cannot allow expansion and space may not be sufficient for the storage of reagents [40••]. There is a lack of established supply chains in resource-limited areas for sequencing reagents, and being a new field of study, even centralized laboratories have supply chain issues and lack MinION-specific sequencing skills [32].

## Opportunities and Improvements that Would Enable Use of MinION WGS in Low-Resource Settings

Simple DNA extractions, like PDQeX DNA purification technology, where DNA is extracted and purified within the same tube system should be encouraged in low-resource settings where it is difficult to establish a well-equipped laboratory for DNA extraction [38, 75]. Design and development of MinION WGS software which works offline without the need of internet will provide greater access to the technology in remote areas where internet connections and stable power supplies are not possible [39]. Sequence modules and online training in bioinformatics are a necessity to conduct successful MinION WGS projects. A 1-month training module for MinION which includes bioinformatics, a software tutorial, and hands-on training on the extraction of DNA/RNA, library preparation, and loading of the samples has been designed which can make technology use possible in low-resource settings [76•]. Yet, in-person or on-site training before a project is conducted would allow users to develop confidence in running and analyzing data [40••, 68]. Online training can also provide alternative opportunities for accessing more modules and techniques, due to their availability to all researchers. Regional and international surveillance should be encouraged more as it provides pooled funding, allows sharing of knowledge and sequencing data, and also encourages monitoring of genetic variations in disease-causing organisms over time [32, 40••]. Additionally, collaborative studies should be encouraged among developed nations and research programs in low-resource settings. These collaborations promote data sharing and also offer necessary training and provide additional funding and sharing of experience from the developed world to resource-limited areas [40••]. Collaborations also provide opportunities for sharing and application of protocols developed for outbreaks of diseases in the developed world to be used in low-resource setting areas [35]. Use of protocols designed in developed nations would enable quick application, especially in outbreak investigations, without the initial cost of development and optimization [39, 40••, 59•, 60••]. Finally, development of field laboratory packages by the manufacturer and project engineers would encourage the use of technology with minimal laboratory infrastructure. For example, equipment and consumables packaged within self-contained systems that also work as temporary bench tops in the field would greatly enhance the accessibility of MinION technology in remote regions of the world (38, 39).

## Conclusion

MinION WGS is becoming a commonly used sequencing method in the developed world, but MinION sequencing is most likely to have the largest impact by providing accessible sequencing capability in low-resource settings. While many protocols to use the MinION sequencing technology are established, these protocols often do not account for the utilization of the technology in low-resource settings. This technology has already shown significant benefits in the context of outbreak investigations and surveillance of drug-resistant isolates. If barriers to accessibility for use in low-resource settings can be overcome, there is potential for vast implementation across the globe that will lead to exciting new research avenues and discoveries.
